# Can probiotics trigger a paradigm shift for cleaning healthcare environments? A narrative review

**DOI:** 10.1186/s13756-024-01474-6

**Published:** 2024-10-08

**Authors:** Luisa A. Denkel, Andreas Voss, Elisabetta Caselli, Stephanie J. Dancer, Rasmus Leistner, Petra Gastmeier, Andreas F. Widmer

**Affiliations:** 1 Institute of Hygiene and Environmental Medicine, Charité Universitätsmedizin Berlin, Corporate Member of Freie Universität Berlin, Humboldt-Universität zu Berlin and Berlin Institute of Health, Hindenburgdamm 27, 12203 Berlin, Germany; 2 National Reference Center for the Surveillance of Nosocomial Infections, Charité Universitätsmedizin Berlin, Corporate Member of Freie Universität Berlin, Humboldt-Universität zu Berlin and Berlin Institute of Health, Berlin, Germany; 3 Department of Medical Microbiology and Infection Control, University Medical Center Groningen, Groningen, The Netherlands; 4 Section of Microbiology, Department of Chemical, Pharmaceutical, and Agricultural Sciences, University of Ferrara, Ferrara, Italy; 5 NHS Lanarkshire & Edinburgh Napier University, Edinburgh, UK; 6 Department of Gastroenterology, Infectious Diseases and Rheumatology, Charité Universitätsmedizin Berlin, Corporate Member of Freie Universität Berlin, Humboldt-Universität zu Berlin and Berlin Institute of Health, Berlin, Germany; 7 Faculty of Medicine, University of Basel, Basel, Switzerland; 8 Swissnoso – Swiss National Center for Infection Prevention, Bern, Switzerland

**Keywords:** Probiotics, Cleaning, healthcare-associated infections (HAI), Antimicrobial resistance genes (ARG), Expert recommendations

## Abstract

**Background:**

The environment of healthcare institutions plays a major role in the transmission of multidrug resistant organisms (MDRO) and likely in subsequent healthcare-associated infections (HAIs). Probiotic cleaning products are a novel option for environmental cleaning. They represent a sustainable and biodegradable alternative to conventional chemical disinfectants for controlling microbial bioburden, and preventing pathogen transmission in hospital environments. High-quality studies including randomized clinical trials (RCT) triggered a summary with expert recommendations until further studies allow a critical review and meta-analysis of the data.

**Methods:**

Infection control experts from five European countries summarized available data as of June 2023. Authors presented their published RCTs, reviewed the existing literature on probiotic cleaning, summarized the results and identified knowledge gaps and subsequent research needs.

**Results:**

Probiotic cleaning was similarly effective for reducing HAI-related pathogens, enveloped viruses such as SARS-CoV-2 and MDRO in environmental samples compared to conventional chemical disinfectants. More importantly, probiotic cleaning was non-inferior to disinfectants in terms of preventing HAI in a large RCT. In addition, probiotic cleaning has also been shown to reduce antimicrobial resistance genes (ARG), costs and antimicrobial consumption in other hospital trials. They are biodegradable, do not require any protection for chemical hazards, and are compliant with occupational health. A paradigm shift, however, requires a very strong evidence to justify for such a change. In the past, this evidence was limited by the heterogeneity of study design, products, protocols, and few studies on clinical outcomes used in the trials. Furthermore, the regulatory, safety, and quality aspects of probiotic cleaning products are not, yet, completely defined and require clearing by authorities.

**Conclusion:**

To date, probiotic cleaning is a breakthrough technology and a biological alternative for chemical disinfectant when treating hospital environment. It may also have a positive effect on MDRO transmission. However, the different compositions of probiotic products will require standardization, and more robust data should be generated to support these promising results on different compositions. This may trigger a paradigm shift in cleaning of healthcare institutions from chemical to biological control of the hospital environment.

**Supplementary Information:**

The online version contains supplementary material available at 10.1186/s13756-024-01474-6.

## Background

Patients in healthcare institutions including hospitals are at risk of acquiring healthcare-associated infections (HAI) and multi-drug resistant organisms (MDRO). Infection control primarily aims to prevent HAIs and MDRO transmission by various measures such as hand hygiene, isolation of MDRO carriers, and decontamination of the hospital environment by surface cleaning and disinfection [1–3]. The coronavirus-disease 2019 (COVID-19) pandemic brought the importance of environmental cleaning and disinfection back to public focus. Further, the number of reports on prolonged contamination of the hospital environment with MDRO, plasmids carrying antimicrobial resistance genes (ARG) and newly emerging pathogens such as *Candida auris* has risen [4, 5]. Gram-positive bacteria such as vancomycin-resistant *Enterocococcus faecium* (VRE) and fungi like *Candida subspecies (ssp.)* can survive on surfaces for days and weeks, and are difficult to eliminate from the environment [6, 7]. Additionally, multidrug-resistant Gram-negative bacteria (MDRGN) producing carbapenemases like New Delhi metallo-beta lactamase (NDM) or Oxacillinases β-Lactamases (OXA) are increasing in European hospitals [8–11]. This increase has been driven by NDM- or OXA-producing *Klebsiella pneumoniae* even leading to severe hospital outbreaks [1, 8, 9, 11]. Patient rooms not properly disinfected may threaten patients by exposure of the residual contaminated patient room without any direct contact [12]. Hands can be equally contaminated by shaking hands or touching surfaces, while hand hygiene often suffers from poor compliance due to several barriers such as insufficient time, high workload, and understaffing [13, 14]. Universal gloving has also failed to reduce acquisition of methicillin-resistant *Staphylococcus aureus* or VRE [15].

Thus, alternatives to current cleaning practices such as automated decontamination devices, UV (ultra violet) light decontamination, novel disinfectants and probiotic-based cleaning has become increasingly attractive to prevent environmental contamination, in particular with MDRO and *Clostridoides (C.) difficile*. New studies provide some encouraging evidence that probiotic-based cleaning have beneficial effects not only on environmental control, but also on HAI and dissemination of ARG. Various probiotic cleaning products are cleared to the market and have been used for animal breeding, catering, cleaning in public buildings and private households [16–20]. However, appropriateness and safety of probiotic products for routine application in hospitals have not, yet, been fully determined, although some studies report promising data on their genetic stability and lack of infectious potential in hospitalized patients [21, 22].

In 2022, the Commission for Hospital Hygiene and Infection Prevention (KRINKO) at the German national public health institute (Robert-Koch Institute, RKI) published new recommendations on cleaning and disinfection of surfaces in healthcare institutions [23]. Use of probiotic cleaning in hospitals was not yet recommended, as the existing evidence was considered insufficient at the time. While chemical disinfection was acknowledged as indispensable, probiotic cleaning was explicitly mentioned as an interesting approach for healthcare institutions. This assessment is justified by the potential of probiotic bacteria to establish a long-term stable microbiome, partly also replacing nosocomial pathogens. Additionally, most disinfectants only have short-term effects, can lead – also rarely - to resistances to disinfectants and cross-resistances to antibiotics, and may pose risks to both humans and the environment [23].

An expert group, mainly authors of publications on this topic, reviewed the current literature to provide a narrative review with emphasis on the most recent randomized controlled clinical trials (see supplemental material for more details). They also focused on regulatory affairs of probiotic cleaning products. Staff from the cleaning services were involved to cover practical aspects and pitfalls for implementation of probiotic cleaning in a hospital. The objectives of this narrative were set as follows:

1. To compare pros and cons of commonly used, commercially available products for environmental cleaning/disinfection in healthcare institutions, in particular detergents, chemical disinfectants and probiotics.
2. To discuss existing literature on probiotic cleaning and positioning of the technology.
3. To share experiences, knowledge and data on probiotic cleaning in different European hospitals.
4. To define knowledge gaps to be addressed by future research.

## Probiotics: concepts and mechanisms

Critical illness is known to be associated with the loss of “health-promoting” commensal microorganisms and the overgrowth of pathogenic bacteria, a process being called dysbiosis [24, 25]. Beneficial effects by a high biological diversity were reported for the human microbiome of patients, but also for the hospital microbiome [26–29]. Disinfectants work not selectively by reducing/ eliminating all microorganisms on hospital surfaces. During re-colonization after disinfection, non-desirable microorganisms such as pathogens and MDRO may benefit from a lower competitive pressure for resources in the habitat. In consequence, they may outcompete harmless microorganisms [23, 30]. In contrast, the principle of probiotics is based on competitive exclusion. As a biological paradigm, species competing for the same limited resources are not able to coexist in the same biological niche [23, 31]. Probiotics colonizing the hospital environment compete with potential pathogens and MDRO for nutrients and the habitat. Some probiotic bacteria can even secrete secondary metabolites that provide them with a survival advantage. Thus, probiotic cleaning offers an eco-friendly and effective method for maintaining cleanliness and hygiene in various healthcare settings by harnessing the natural competition between beneficial bacteria and harmful pathogens.

Mostly, probiotic cleaning products not only contain probiotic species, but also prebiotics for growth promotion (e.g. inulin) and detergents. Commonly used microorganisms for probiotic formulations are *Bacillus subspecies* (ssp.), *Lactobacillus* ssp., *Streptococcus* ssp., *Bifidobacterium* ssp. and the yeast *Saccharomyces* ssp [1, 12]. The mechanisms resulting in probiotic cleaning effects have not been fully ascertained. The technical concepts are envisioned as follows: (i) probiotic cleaning regimens use beneficial microorganisms to clean surfaces in accordance with their biological and metabolic activities; (ii) Probiotic strains inhibit expansion and survival of potential harmful viable pathogens by competitive exclusion [1, 13, 14]. This can be achieved by direct or indirect mechanisms including lowering pH, generating compounds that inhibit the growth of pathogens or compete for nutrients [12, 13, 15]. Beyond that, probiotic strains can produce extracellular enzymes (e.g. proteases, cellulases, amylases, ureases) that are capable of metabolizing and degrading dirt, food and soil. Metabolites generated by protein degradation, fatty acids and other organic compounds can be further broken down into smaller products. These processes might be advantageous for odour control, e.g. by preventing their production or by disrupting odour-intense compounds like H_2_S or NH_3_ [12]. Some probiotic-based formulations appear to have long-lasting effects. This is shown by spore-forming bacteria like *Bacillus* spp. that remain on surfaces even after the cleaning process has been completed. Spores have the potential to germinate, which provides vegetative cells that are able to proliferate and prevent re-contamination by harmful microorganisms [12, 15]. This reduces the overall pathogenic bioburden and can even target MDROs. Furthermore, some probiotic species might be able to hinder pathogens from forming biofilm by reducing pathogen adhesion, co-aggregation, disrupting cell metabolism and/or interfering with quorum sensing [13].

## Summary of studies on probiotics for environmental cleaning in hospitals

### Reviewing existing data on probiotic cleaning in hospitals

The results of published cleaning intervention trials using probiotics are summarized in Table 1.

**Table 1:** Overview of publications on cleaning intervention trials included in this narrative review. CFU, colony forming units., PCHS, Probiotic Cleaning Hygiene System. spp., subspecies

No.	Author, Year, Journal, publication year	(Probiotic) intervention	Duration of intervention	Control group	Setting, country	Study design	Sample size	Outcome	Results
1	Vandini, Plos One, 2014 [38]	Probiotic Cleaning Hygiene System (PCHS; Copma srl, Italy), by using detergents containing 10^7^ spores per ml of *B. subtilis*, *B. pumilus* and *B. megaterium* (Chrisal, Lommel, Belgium)	24 weeks	Conventional cleaning: chemical detergents in 1 Belgian hospital (Ecolab, Groot-Bijgaarden, Belgium), In both Italian hospitals: chlorine-based detergents (Actichlor for all washable surfaces, Diversey S.p.A., Italy)	3 hospitals, Belgium (*n* = 1), Italy (*n* = 2)	Multicentre, pre-post interventional study	20,000 environmental samples	Presence and survival of several microorganisms responsible for HAIs (i.e. coliforms, *Staphylococcus aureus*, *Clostridiodes difficile*, and *Candida albicans*) on hard surfaces by CFU / m²	Reduction of HAI-related pathogens by 50 to 89%
2	Caselli, Plos One, 2016 [22]	See publication no. 1	6 months	No control group	1 hospital, Italy	Single centre, pre-post interventional study	360 environmental samples collected in duplicates (*n* = 720)	Growth of *Staphylococcus spp*. and *Staphylococcus aureus*, *Enterobacterales*, *Acinetobacter spp.*, *Pseudomonas spp*., *C. difficile*, *Candida spp.* and *Aspergillus spp*. by CFU or genome copies/ m²; Spore germination (in vitro), antibiotic resistance genes	Decrease of the number of CFU/m^2^ for all pathogens tested except for Enterobacterales due to scarce presence at T0, Germination of *Bacillus* spores on dry inanimate surfaces demonstrated (*in vitro*), Decrease of antibiotic resistance genes in the contaminating microbial population
3	Caselli, Plos One, 2018 [35]	See publication no. 1	6 months	Conventional chemical-based chlorine products	6 hospitals, Italy	Multicentre, pre-post interventional study	11,842 patients and 24,875 environmental samples	Qualitative and quantitative characterisation of hospital surface bioburden	Significant decrease of HAI cumulative incidence from a global 4.8% (284 patients with HAI over 5,930 total patients) to 2.3% (128 patients with HAI over 5,531 total patients) (OR = 0.44, CI 95% 0.35 ± 0.54) (*P* < 0.0001), 2 Log drop of surface microbiota drug-resistance genes (*P* < 0.0001; *Pc* = 0.008)
4	Caselli, Infection and Drug Resistance, 2019 [34]	Additional analysis of trial no.3 [22]	6 months	Conventional chemical-based chlorine products	5 hospitals, Italy	Multicentre, pre-post interventional study	756 environmental samples, antimicrobial drug consumption analysis and economic analysis based on 398 patients with HAI and data available	Surface microbiota, AMR (by microarray, nested PCR, antibiogram, and microdilution tests), antibiotic consumption and associated costs	Up to 99% decrease of the AMR genes harbored by surface hospital microbiota, Antimicrobial drug consumption associated with HAI onset showed a global 60.3% decrease, with a 75.4% decrease of the associated costs.
5	Tarricone, Pathogens 2020 [33]	Economic analysis of trial no.3 [22], conventional chemical cleaning (CCC) versus PCHS	6 months	Conventional chemical-based chlorine products	6 hospitals, Italy	Economic analysis of trial no.3 [22]: Multicentre, pre-post interventional study with subsequent propensity-score matching (estimated increase of PCHS use from 5–50% in the next 5 years),	11,461 patients (propensity score matching)	Number of HAIs per 1000 patient days in both CCC and PCHS periods; HAIs outcomes; Consumption of drugs to treat HAIs and the related costs; Identification of cases of antibiotic-resistance and estimation of their treatment costs	Cumulative incidence of HAI: 4.6% and 2.4% (*p* < 0.0001) for CCC (*N* = 4160) and PCHS (*N* = 4160), (OR = 0.47, CI 95% 0.37–0.60), with severe HAIs of 1.57% vs. 1% and antibiotic resistances of 1.13% vs. 0.53%, Estimated savings by PCHS: about 31,000 HAIs and 8,500 antibiotic resistances, 14 million euros, of which 11.6 for the treatment of resistant HAIs
6	Soffritti, Infect Drug Resist 2022 [37]	See publication no. 1	2 months	Conventional chemical-based chlorine products	1 hospital (Maternal and Child Health Institute), Italy	Single centre, pre-post interventional study	152 samples environmental samples	Microbial contamination (monitored by culture-based CFU count, 16 S rRNA NGS for bacteriome characterization and microarrays for resistome assessment), SARS-CoV-2 presence (monitored by PCR)	PCHS usage was associated with a stable 80% decrease in surface pathogens compared to levels detected for chemical disinfection (*P* < 0.01), up to 2 log decrease in resistance genes (*P* < 0.01) SARS-CoV-2 was not detectable in the pre-PCHS and PCHS period.
6	D’Accolti Microbiome 2023 [46]	PCHS (prepreg cloths/ nebulization) was applied 30 min after ethanol/ammonium disinfection (see publication no. 1) versus chemical disinfection with chlorine-, ethanol-, and ammonium salt-based products	12 weeks	Routine chemical disinfection with chlorine-, ethanol-, and ammonium salt-based products	Two underground driverless trains with four compartment each corresponding to a total of 96 seats and 438 standing places for a total 50.5 m in length and 73 m^2^ of surface. 1 train with PCHS, 1 train with standard disinfection	Non-randomized, controlled interventional trial	6 sampling campaigns, sampling of 12 total points in duplicates from surfaces (floor, seats, handrails, doors) and air filters (if available), collected by RODAC plates /sterile swabs	Microbial growth (culture-based CFU count), 16SrDNA NGS, real-time qPCR microarray, NGS profiling	Clear and significant decrease in bacterial and fungal pathogens (*p* < 0.001), decrease of SARS-CoV-2 presence (*p* < 0.01) in the probiotic-treated train compared with the chemically disinfected control train
7	Klassert Clin Microbiol Infect 2022 [28]	0.5% detergents with concentration containing 10^7^ spores per ml *B. subtilis* (ATCC6051), *B. megaterium* (ATCC14581), *B. licheniformis* (ATCC12713), B. *pumilus* (ATCC14884) ,and *B. amyloliquefaciens* (DSL13563-0) (by Chrisal)	3 months	Soap-based arm: agent with non-ionic surfactants, anionic surfactants, and fragrances in a total concentration of 1% (Brial Top^®^, Ecolab Inc.). Disinfectant arm: 2-phenoxyethanol (10%), 3-aminopropyldodecylamine (8%), benzalkonium chloride (7.5%) at a total concentration of 1.5%, with a contact time of 15 min (Incidin Pro^®^, Ecolab Inc.)	1 hospital, Germany	Single centre, pre-post interventional study	1,019 environmental samples for analysis of environmental microbiota 551 environmental samples for ARG detection	Environmental microbiota (by 16 S rRNA sequencing), Detection of antibiotic resistance genes (ARGs) by multiplex Taq-Man qPCR assays	Significant increase in the alpha-diversity metrics samples from the floor (*p* < 0.001) and the sink (*p* < 0.01), but not from door handles during the probiotic strategy (compared with disinfectant), Displacement of the intrinsic environmental microbiota in sink samples (median 16 S-rRNA copies = 138.3; IQR: 24.38–379.5) compared to traditional disinfection measures (median 16 S rRNA copies = 1343; IQR: 330.9–9479; *p* < 0.05). Significant reduction in the total ARG counts in the sink samples during probiotic cleaning (mean ARGs/sample: 0.095 ± 0.067) compared to the disinfection strategy (mean ARGs/sample: 0.386 ± 0.116; *p* < 0.01)
8	Leistner, eClinicalMedicine, 2023 [32]	0.5% detergents containing 10^7^ spores per ml *B. subtilis* (ATCC6051), *megaterium* (ATCC14581), *B. licheniformis* (ATCC12713), *B. pumilus* (ATCC14884), and. *amyloliquefaciens* (DSL13563-0) (by Chrisal)	4 months	Soap-based arm: agent with non-ionic surfactants, anionic surfactants, and fragrances in a total concentration of 1% (Brial Top^®^, Ecolab Inc.). Disinfectant arm: 2-phenoxyethanol (10%), 3-aminopropyldodecylamine (8%), benzalkonium chloride (7.5%) at a total concentration of 1.5%, with a contact time of 15 min (Incidin Pro^®^, Ecolab Inc.)	1 hospital, 18 wards, Germany	cRCT with cross-over design	13.896 patients from 18 wards	Incidence density of HAI Incidence density of HAI with MDRO	Incidence density of HAI was similar between the reference group (soap), disinfectant and probiotics: 2.31 cases per 1000 exposure days for soap, 2.21 for disinfectant (IRR 0.95; 95% CI 0.69–1.31; *p* = 0.953) and 2.21 for probiotics (IRR 0.96; 95% CI 0.69–1.32; *p* = 0.955). Incidence density of MDRO infection was similar between the reference group (soap), disinfectant and probiotics: 0.53 cases per 1000 exposure days (0.32–0.84) for soap versus 0.49 (0.29–0.78) for disinfectant and versus 0.46 (0.26–0.76) for probiotics
9	Dancer, BMC Med 2009 [52]	Enhanced cleaning with CINCH™ detergent (AGMA, Haltwhistle, UK) and water and Tuffie™ wipes (Health Care Services, Nottinghamshire, UK) by an additional cleaner	6 months per phase	Conventional cleaning with the same detergent and wipes	1 hospital; 2 wards, UK	Single-center, prospective cross-over trial	Environmental samples from 10 hand-touch sites (e.g. infusion pump, blood pressure, and computer keyboard) on both wards every week for 1 year	Microbial contamination on hand-touched sites, Incidence MRSA infections, Cost savings	Reduction of microbial contamination at hand-touched sites by 32.5%, Reduced the number of new MRSA infections by 26.6%, Cost savings between 30.000 and 70.000 pounds estimated

At the Charité University Medicine hospital in Berlin, Germany, two trials were conducted with the probiotic cleaning product SYNBIO^®^ (HeiQ Chrisal NV, Lommel, Belgium) containing five different *Bacillus* species, i.e. *B. subtilis*, *B. megaterium*, *B. licheniformis*, *B. pumilus* and *B. amyloliquefaciens*. In the first trial, one neurological ward was subsequently cleaned with the probiotic product, detergents or disinfectants (for 3 month each) [28]. The study showed significant increases in biological diversity metrics (alpha-diversity) compared with disinfection in the floor (*p* < 0.001) and the sink samples (*p* < 0.01). For the door handle samples, however, alpha-diversity was significantly more diverse (*p* < 0.05) for detergents (compared with disinfection). Further, the probiotic cleaning product reduced the occurrence of *Pseudomonas* spp. in environmental samples compared with chemical disinfection [28]. In addition, the study also demonstrated a reduction of antimicrobial resistance genes (ARG) in environmental samples after cleaning hospital rooms with probiotic cleaning products compared with chemical disinfectants [28], in particular mecA resistance genes present in methicillin resistant *Staphylococcus aureus* (MRSA) [28]. The second study focused on the questions whether these effects on the hospital environment may translate into clinically relevant outcomes such as HAI or MDRO incidence. The study question was addressed by a cluster randomized controlled trial (cRCT) with cross-over design conducted in 18 non-intensive care units (non-ICUs) [32]. Disinfectants, detergents and probiotics were similarly effective for environmental cleaning as well as preventing HAI or HAI with MDRO [32].

In one Belgian and five Italian hospitals, a probiotic cleaning hygiene system (PCHS^®^, Copma scrl, Ferrara, Italy) was introduced [22, 33–38]. This probiotic-based sanitation is a cleaning procedure involving a probiotic product provided by HeiQ Chrisal NV (Lommel, Belgium) with three *Bacillus* species (*B. subtilis*, *B. pumilus* and *B. megaterium*) as previously described [38]. In hospital environmental samples, PCHS significantly reduced the abundance of HAI-related pathogens [22, 37, 38] and the presence of ARG [22, 34, 35, 37] compared with chemical disinfection. Parallel independent studies confirmed the reduction of contamination with pathogenic microorganisms [39, 40]. Another Italian trial conducted in a children’s hospital emergency ward suggested that PCHS cleaning was as effective as chlorine-based chemical disinfection for elimination of Severe acute respiratory syndrome coronavirus 2 (SARS-CoV-2). This enveloped virus was neither detectable after PCHS cleaning nor after chemical disinfection with chlorine [37]. However, it has been shown that SARS-CoV-2 can also be effectively removed from hard, non-porous surfaces by hard-water damped wiping only [41]. PCHS was associated with a significant reduction of cumulative HAI from 4.8 to 2.3% (OR = 0.44; CI95% 0.35–0.54), a result not observed in the German cRCT [35]. Such considerable impact of PCHS may not be reproduced in other settings as limited proportion of HAI are considered preventable [42, 43]. A systematic review and meta-analysis calculated the preventable proportion of HAI to be 35 – 55% with data from 2005 to 2016 in different economic settings [43]. Similar estimations from Germany vary between 13% and 45% of HAI [42]. Antimicrobial consumption and costs were further analysed in two studies [33, 36]. PCHS saved more than 60% of HAI-related antibiotic consumption and more than 70% of associated costs [36]. These results, however, are based on 398 patients with HAI selected from the before-after trial by Caselli et al. [35]. Tarricone and colleagues estimated that 14 million Euro might be saved if PCHS use was increased from 5 to 50% over a period of five years [33]. However, the expert group questioned that these results are transferable to other hospitals given the different hospital settings, study designs, control groups and interventions applied. The German trial used a more robust study design (cross over cRCT) but was a single-center study. In contrast, the Italian trial used a study design more prone to bias (before-after design), but was multi-centered. Cleaning protocols and disinfectants varied with 2-phenoxyethanol, 3-aminopropyldodecylamine, benzalkonium chloride (Indicin Pro^®^) in Germany versus chlorine-products in Italy. Most importantly, the German trial was conducted in a setting with 1.6% HAI incidence compared to the Italian trial with 4.6%. However, both products were provided by the same company. The probiotic product used in the German trial contained five different *Bacillus* species (*B. subtilis* (ATCC6051), *B. megaterium* (ATCC14581), *B. licheniformis* (ATCC12713), *B. pumilus* (ATCC14884) and *B. amyloliquefaciens* (DSL13563-0)). For the Italian trial, a patented cleaning concept (PCHS^®^) was implemented that included probiotic detergent with three *Bacillus* species (*B. subtilis*, *B. pumilus* and *B. megaterium*). Further, the duration of intervention was different (4 months in the German trial versus 6 months in the Italian trial), as well as timing of sampling. In the Italian trial, sampling was always performed seven hours after cleaning (thus allowing recontamination) whereas in the German study the sampling time was variable and resulting data could be affected by residual action of disinfectants in the chemical sanitation arm. Furthermore, the German study results might be limited by the fact that probiotic cleaning was interrupted by terminal and / or targeted disinfection, in particular if patient rooms were occupied with carriers of MDROs or other notifiable pathogens. Emergency chemical disinfection also occurred in the Italian studies, where only continuous usage of sporicidal disinfectants was shown to prevent probiotic sanitation effects [44, 45]. A limitation that might have occurred in both trials was cross-contamination by shoes or hands of healthcare workers between study arms or wards participating in the trials and those not.

Hospital cleaning is considered an important part of infection control [29, 46–49]. Appropriate cleaning practices require a number of careful decisions, e.g. cleaning frequencies, materials, techniques, equipment and agents used as well as identification of critical- und non-critical areas. At the same time, the amount of research on basic cleaning is limited [29]. Evidence-based decision making in this field is challenging as, to date, there is no standard methodology for measuring microbial bioburden on surfaces, nor are there international benchmark standards for surface bioburden levels indicating potential infection risks [29, 50]. Indeed, understanding the difference between the terms ‘cleaning and ‘cleanliness’ remains an issue and is crucial when sharing expertise on this topic. While ‘cleaning‘ represents the physical process of removing surface soil, ‘cleanliness‘ is defined as residual soil on surfaces after the cleaning process [29]. Assessment of cleanliness is possible by identification and quantification of indicator organisms that pose a high risk to patients (< 1 cfu/cm²) such as *C. difficile* or *S. aureus*. This can be performed alongside quantitative assessment of organisms on hand-touch sites using microbiological sampling (< 2.5–5.0 cfu/cm²) or adenosine triphosphate (ATP) counts using ATP bioluminescence systems as surrogate markers for bioburden [48, 50]. In contrast, fluorescent markers and ATP bioluminescence systems as well as direct supervision, observation and education of housekeeping staff are used to monitor the cleaning process [48]. Both surrogate markers do not necessarily correlated with the bioburden. A prospective cross-over trial conducted on two hospital wards in the United Kingdom (UK) demonstrated that enhanced cleaning was associated with a reduction of microbial contamination at hand-touch sites by 32.5% and reduced the number of new MRSA infections by 26.6% [51]. Cost savings were estimated between 30,000–70,000 pounds in this trial [51]. Enhanced cleaning was performed with detergents and included one additional cleaner per ward who focused on high-touch surfaces such as door handles, infusion pumps and computer keyboards. Disinfectants were not routinely used on these wards other than bleach (sodium hypochlorite) for bathrooms [51]. Thus, enhanced cleaning without changing any substances but increasing staff and cleaning frequencies reduced more than 25% of new MRSA infections compared with the standard cleaning protocol [51]. This supports the argument that physical removal of surface bioburden might be more important than the substances applied. It is possible that microfiber and water by themselves may be sufficient for routine cleaning in most cases. Despite the variations of the probiotic trials discussed above, there is consensus that probiotic cleaning was non-inferior compared with disinfectants in both trials [32, 35].

### What is the added value of probiotic cleaning for the decontamination of the hospital environment?

Many healthcare institutions still do not prioritize environmental cleaning as essential measure for patient safety [52]. The impact of environmental control on HAI incidences is difficult to assess, as multiple factors such as failure with hand hygiene, susceptible patients, and infectious material (inoculum) are required to induce infection. Recently, awareness for this topic has grown due to an increasing number of studies that link interventions in the hospital with lower HAI rates and/ or patient colonization [52]. Further, a RCT emphasized the importance of cleaning / disinfecting the hospital room before the next patient is admitted [12].

Some evidence exists on the fact that probiotic cleaning may have additional benefits concerning sustainability, cost-effectiveness, occupational safety, sustaining a biologically diverse hospital microbiome, and odor control, compared with chemical disinfection. Chemical disinfectants have been used for decades, especially in high-risk areas such as intensive care units (ICUs). Even highly effective substances were shown to have limited impact, as re-colonization rapidly occurred after disinfection [53]. MDROs were found in dry surface biofilms from ICU surfaces despite terminal cleaning with disinfectants, e.g. with chlorine solution [54, 55]. Similarly, terminal chemical disinfection is frequently insufficient to eradicate *Candida auris* [4].

It is not known whether probiotic cleaning might be an adequate supplement to fill this gap as suggested by some trials analysing environmental samples [22, 28, 35], or whether additional procedures such as UV decontamination are required to safely remove MDROs.

Heavy and repetitive use of antiseptics and disinfectants are associated with reduced tolerances of clinical isolates to these agents, development of cross-resistance to antibiotics and other potentially detrimental effects on health and environment [1, 56, 57]. As an example, emergence of resistance to glutaraldehyde has been observed [58]. Another worrisome trend is the occurrence of cross-resistances among disinfectants and antimicrobials [59]. In addition, chemical disinfectants are harmful to the environment and their handling is potentially hazardous to health of cleaning staff and healthcare workers. More specifically, currently used chemical disinfectants such as glucoprotamin, aldehydes, and quaternary ammonium compounds may form phenolics and aldehyde toxic fumes that are problematic for health and the environment. Further, the use of glucoprotamin in its concentrated form requires specific carefulness and personal protective equipment by hospital staff [60].

Therefore, new technologies and compounds are required to add to the currently available disinfectants that are at least equally effective, but less harmful to the environment and wellbeing of healthcare workers and cleaning staff.

Advantages and disadvantages of three cleaning regimens - detergents, disinfectants and probiotics - identified by the expert group are summarized in Table 2. The most important priority is patient safety, but other aspects such as sustainability, costs, occupational safety, effects on the environmental microbiome and applicability must also be considered and weighed up against each other.

**Table 2:** Characteristics of cleaning regimes stratified by advantages (pro) and disadvantages (contra) as discussed during the workshop. Results presented here represent consensus of all workshop participants

Topic	Detergents (Soap and water)	Disinfectants	Probiotics
Environmental safety / sustainability	Some products (not all) can be biodegradable, sustainable and environmental friendly (PRO / CONTRA)	Not biodegradable, not environmental friendly, not sustainable (CONTRA)	Biodegradable, environmental friendly, sustainable (PRO)
Occupational safety	No dangerous substance, usually harmless for occupational health, allergies possible (PRO)	Dangerous substance, potentially harmful for occupational health, allergies possible (CONTRA)	No dangerous substance, harmless for occupational health, allergies possible (PRO)
Is the method well established?	Very well established (PRO)	Very well established, critical values for reduction / elimination of microorganisms available (PRO)	Not well established, future studies and standardization needed (CONTRA)
Regulations	Meet current regulations / recommendations / national and international guidelines (PRO)	Meet current regulations / recommendations / national and international guidelines (PRO)	Do not meet current regulations / recommendations / national and international guidelines, new regulations necessary, currently only available in Europe (CONTRA)
Costs	Low costs (PRO)	High costs (CONTRA)	Higher costs, potentially additional costs by quality control measures and monitoring of potential side effects (CONTRA)
Antiseptic / antimicrobial resistances	No effect on antiseptic / antimicrobial resistances to be expected (PRO/CONTRA)	Has the potential to increase antiseptic / antimicrobial resistances (CONTRA)	Might prevent antimicrobial resistance (PRO)
Longterm cleaning effect	No long-lasting cleaning effect (CONTRA)	No long-lasting cleaning effect (CONTRA)	Longer lasting effects for days, more effective in removal of organic pollution on surfaces (PRO)
Quality control / monitoring	Quality control and monitoring activities established, no additional activities are necessary (PRO)	Quality control and monitoring activities established, no additional activities are necessary (PRO)	Quality control and monitoring necessary (might generate additional costs) (CONTRA)
Risk of contamination	Moderate risk of contamination by other bacteria, e.g. with Gram-negatives (PRO)	Very low risk of contamination by other bacteria, e.g. with Gram-negatives (PRO)	Risk of contamination by other bacteria due to prebiotics (e.g. inulin), e.g. with Gram-negatives (CONTRA)
Effect on the diversity of the hospital microbiome	No negative effect on the diversity of the hospital microbiome (PRO)	Reduces diversity of the hospital microbiome (CONTRA)	May reduce the biological diversity of the hospital microbiome, but shifts the balance towards beneficial microorganisms (PRO)
Universal applicability	Not adequate for rooms that needs to be sterile (e.g. operating theatre) (CONTRA)	Adequate for rooms that needs to be sterile (e.g. operating theatre) (PRO)	Not adequate for rooms that needs to be sterile (e.g. operating theatre) (CONTRA)
Additional risks by living organisms	None (PRO)	None (PRO)	Potential of living organisms to take up genes (e.g. antimicrobial resistance genes). Available data show high genetic stability and lack of infectious risk in hospitalized patients but they need further confirmation. (CONTRA)

### Defining knowledge gaps that need to be addressed by future research

This narrative review does not discuss bacteriophage preparations such as probiotic-phage sanitation (PCHSϕ) [61]. The latter contains probiotic detergents and bacteriophage preparations (e.g. a mixture of selected lytic phages directed against *Staphylococcus* spp., *Streptococcus* spp., *Proteus* spp., *E. coli* and *Pseudomonas (P.) aeruginosa*) that are commercially available by the Eliava Institute (Staphylococcal phage and Pyophage; GA, USA). This is beyond the scope of this work, as clinical outcome studies are not yet published.

Some trials showed the effect of probiotic cleaning products against enveloped viruses such as SARS-CoV-2 in controlled laboratory conditions, in hospital (emergency room of a children’s hospital) and non-hospital settings [37, 45, 62]. However, data on non-enveloped viruses, e.g. noroviruses, are lacking.

Another question concerns the best composition of probiotic detergents. Various in vitro studies show that probiotic species such as *Bacillus* and *Lactobacillus* spp. may be used for biofilm control of relevant pathogens in hospitals including *Enterococcus faecium*, *S. aureus*, *Klebsiella pneumoniae*, *Acinetobacter baumannii*, *P. aeruginosa*, *Enterobacter* species, and *Escherichia coli* [63]. Molecular analyses revealed that probiotic-based products reduced the antimicrobial resistance (AMR) related gene expression in *K. pneumoniae*, but not in *A. baumanii* [1]. Another in vitro study compared hospital surfaces that were treated for eight months either with disinfectants (3.5% sodium hypochlorite), soap (saponified vegetable extract, essential oils, natural gum) or a probiotic cleaner (Bacterrorist non-toxic all-purpose cleaner) containing spores of *Bacillus spp.* [64]. Subsequently, in vitro experiments investigated whether the “resident microbiome” established during the 8-months-cleaning regimens with either disinfectants, soap or a probiotic cleaner could be overwhelmed by the pathogens *E. coli*, *S. aureus* and biofilm-generating *P. aeruginosa*. Resident microbiomes of surfaces treated with soap and probiotic cleaning but not disinfectants successfully outcompeted *E. coli* and *S. aureus*. At the same time, the resident microbiome overwhelmed *P. aeruginosa* on surfaces treated with soap, while the resident microbiome on surfaces treated with probiotic cleansers failed to completely replace *P. aeruginosa*. Thus, not only the mass of microbial cells but also a higher diversity of microbial species seems to be critical to outcompete certain pathogens including biofilm-forming *P. aeruginosa.* The resident microbiome on surfaces treated with disinfectants (sodium hypochlorite) were totally overwhelmed by biofilm-forming *P. aeruginosa* [64].

Routine and widespread application of probiotic cleaning products in hospitals require regulations, safety standards and quality controls that need to be determined, followed and monitored by public authorities to ensure patient safety. Such quality regulations and their clearance by public authorities are essential on the international, but also on the national level. They could represent a crucial step to overcome hurdles that currently prevent this novel option from achieving its breakthrough. These regulations must be realistic and safe, but flexible enough to enable further innovation. The European Union (EU) has already addressed products containing microorganisms, i.e. probiotics, in its “Proposal for a regulation of the European Parliament and of the Council on detergents and surfactants, amending Regulation (EU) 2019/1020 and repealing Regulation (EC) No 648/2004 (COM(2023)217) [65]. Herein, the authors determine that microorganisms intentionally added to detergents, “shall have an American Type Culture Collection (ATCC) number, belong to a collection of an International Depository Authority (IDA) or have had their DNA identified in accordance with a “Strain identification protocol” (using 16S ribosomal DNA sequencing or an equivalent method) […]” [65]. It should be noted that methods applied for strain identification in this context such as 16S ribosomal DNA sequencing must have sufficient accuracy to discriminate between bacterial species. It is not sufficient to aim at the genus level as different species of the same genus can be highly diverse. In general, all living organisms added to detergents that are used in healthcare environments including hospitals must be well characterized preferably by whole genome sequencing.

Despite the fact that some trials demonstrated the reduction of ARG in environmental samples after probiotic cleaning compared with disinfectants [22, 28, 35], there is no evidence for the reduction of newly acquired MDRO by patients after probiotic cleaning [32]. Thus, the potential of probiotic cleaning to reduce antimicrobial resistance genes in the environment, newly acquired MDRO and HAI among patients needs to be addressed in future research. Such trials need to be sufficiently powered, use a robust study design and should, if possible, also include conventional detergent as a control group [29].

## Conclusions

In conclusion, probiotic cleaning is a promising and innovative technology to treat healthcare environments. Current data provide strong evidence to continue researching on probiotic cleaning and gather practical experiences. It has been found to increase biological diversity, reduce the occurrence of certain pathogens, and decrease the presence of ARG in environmental samples. However, the evidence is still not yet sufficient for a paradigm shift to its routine use in hospitals. Currently, the products may show additional benefits at similar cost in areas, where environmental cleaning with detergents was standard of care. High-quality, multi-national, multi-center RCTs with sufficient statistical power are necessary to evaluate the effect of probiotic cleaning on HAI and MDRO acquisition in hospitals. Effectiveness and sustainability are crucial considerations for any new cleaning practices in healthcare institutions. These practices should align with the overall goal of preventing HAIs and controlling the spread of MDRO. While this narrative review summarizes and evaluates the current evidence, further studies and meta-analyses are needed to definitively determine if and where probiotic cleaning can replace or supplement conventional chemical disinfection. Fully understanding the benefits and limitations of probiotic cleaning is essential before considering a paradigm shift toward this environmentally safe approach for cleaning healthcare environments.

## Electronic supplementary material

Below is the link to the electronic supplementary material.

Supplementary Material 1

## Data Availability

No datasets were generated or analysed during the current study.

## Declarations

### Use of artificial intelligence (AI) technologies

Microsoft Copilot (2024) was used to write the abstract and conclusion of this narrative review. Subsequently to using these technologies, the first author reviewed and edited the content as needed and takes full responsibility for this work. All authors have reviewed and edited all versions generated by the first author.
