# Supplementary material for: Can probiotics trigger a paradigm shift for cleaning healthcare environments? A narrative review
Source: Antimicrob Resist Infect Control. 2024 Oct 8;13:119. doi: 10.1186/s13756-024-01474-6 (PMC11462747; doi:10.1186/s13756-024-01474-6)
Supplement: Supplementary file 1 — Supplementary Material 1 [file 13756_2024_1474_MOESM1_ESM.docx]

**Supplementary material**

**Expert group:**

**Andreas F. Widmer**, Swissnoso - National Center for Infection Prevention (Switzerland), Bern, Switzerland and University of Basel, Basel, Switzerland

**Petra Gastmeier**, Institute of Hygiene and Environmental Medicine and National Reference Center for the Surveillance of Nosocomial Infections,Charité Universitätsmedizin Berlin, corporate member of Freie Universität Berlin, Humboldt-Universität zu Berlin and Berlin Institute of Health, Berlin, Germany.

**Rasmus Leistner**, Institute of Hygiene and Environmental, National Reference Center for the Surveillance of Nosocomial Infections and Department of Gastroenterology, Infectious Diseases and Rheumatology, Charité Universitätsmedizin Berlin, corporate member of Freie Universität Berlin, Humboldt-Universität zu Berlin and Berlin Institute of Health, Berlin, Germany.

**Luisa A. Denkel**, Institute of Hygiene and Environmental Medicine and National Reference Center for the Surveillance of Nosocomial Infections,Charité Universitätsmedizin Berlin, corporate member of Freie Universität Berlin, Humboldt-Universität zu Berlin and Berlin Institute of Health, Berlin, Germany.

**Andreas Voss**, Department of Medical Microbiology and Infection Control, University Medical Center Groningen, Groningen, The Netherlands

**Elisabetta Caselli**, Section of Microbiology, Department of Chemical, Pharmaceutical, and Agricultural Sciences, University of Ferrara, Ferrara, Italy

**Stephanie Dancer**, NHS Lanarkshire & Edinburgh Napier University, Edinburgh, United Kingdom.

**Consultants:**

**Robin Temmerman**, CEO/CTO HEIQ Chrisal, Lommel, Belgium.

**Carlo Centonze**, Group CEO HEIQ, Schlieren (Zurich), Switzerland

**Gregor Zakonsky**, Charité Facility Management (CFM), Head of the cleaning department, Berlin, Germany

**Questions being discussed in the group of experts:**

(I) Barriers for implementation:

- What barriers did you experience during implementation of probiotic cleaning among patients, cleaning staff, healthcare workers, hospital management / leaders?
- Which wards/departments should not use probiotic cleaning? (e.g. ICU, operating room)
- Are there possible negative health and environmental impacts which require attention?
- Should probiotic cleaning also be recommended if no reduction of HAI rates can achieved?
  (non inferiority compared with disinfectant)

(II) The substance

- Did the probiotic products used in the Italian trials (patented system by Copma) differ from the products applied in the German trial?
- Are there any other companies that supply probiotic cleaning products?
- If yes, what probiotic species are used?
- Are there any quality criteria that must be met by probiotic cleaning products (prevention of contamination / licensed as detergent in EU?)
- Which regulations have to be considered?
- Which level of microbial diversity is optimal for probiotic cleaning products?

(III) Future research

- Multi-center RCT in ICUs?
- National / international
- Influence of seasons, occupancy, human activities?
- What are the best endpoints for trials on probiotic cleaning products?
- What is the effect of terminal cleaning with disinfectants?
- How long does it take until re-establishment of the probiotic effectiveness?
- What is the impact on probiotic cleaning on different HAI types?
- Does probiotic cleaning have any long term effects?
- Cost effectiveness?
- What about the combination with phages?
